# Supplementary material for: Medical cannabis use in Thailand after its legalization: a respondent-driven sample survey
Source: PeerJ. 2022 Jan 11;10:e12809. doi: 10.7717/peerj.12809 (PMC8759353; doi:10.7717/peerj.12809)
Supplement: Supplemental Information 1 [file peerj-10-12809-s001.pdf]

แบบสอบถามเชิงปริมาณ

สถานการณ์ปัจจุบันของการใช้กัญชาทางการแพทย์ในประเทศไทย

จำนวนเครือข่ายที่ใช้กัญชาทางการแพทย์ที่ท่านรู้จัก..................................คน

ส่วนที่ 1 ลักษณะประชากร

1. เพศ [ ] 1.ชาย [ ] 2.หญิง
2. อายุ ....ปี 2.1 วัน/เดือน/ปี (พ.ศ.) เกิด ....
3. ศาสนา [ ] 1.พุทธ [ ] 2.คริสต์ [ ] 3.อิสลาม [ ] 4.อื่นๆ (ระบุ)..........................
4. วุฒิการศึกษาที่สำเร็จสูงสุด
   [ ] 1.ไม่เคยเรียน [ ] 2.ประถมศึกษา [ ] 3.มัธยมศึกษาตอนต้น [ ] 4.มัธยมศึกษาตอนปลาย
   [ ] 5.อาชีวศึกษา (ปวช./ปวส.) อนุปริญญา [ ] 6.ปริญญาตรีหรือสูงกว่า [ ] 7.อื่นๆ ระบุ ....
5. อาชีพประจำที่มีรายได้หลัก
   [ ] 1.ว่างงานไม่ได้ประกอบอาชีพ [ ] 2.นักเรียน/นักศึกษา [ ] 3.รับจ้างแรงงาน [ ] 4.ทำนา/ทำไร่/ทำสวน
   [ ] 5.ค้าขาย [ ] 6.พนักงาน ลูกจ้างบริษัทเอกชน [ ] 7.รับราชการ
   [ ] 8.พนักงานรัฐวิสาหกิจ [ ] 9.ธุรกิจส่วนตัว/เจ้าของกิจการ
   [ ] 10.พระภิกษุ สามเณร/ชี บาทหลวง อิหม่าม อุลามาอ์ [ ] 11.อื่นๆ ระบุ.............................
6. สถานภาพการสมรส
   [ ] 1.โสด [ ] 2.แยกกันอยู่ [ ] 3.หม้าย [ ] 4.หย่าร้าง [ ] 5.สมรส

7. รายได้เฉลี่ยต่อเดือน .... บาท

8. ภูมิลำเนาที่อยู่ในปัจจุบัน จังหวัด ....

ส่วนที่ 2 ประวัติการใช้กัญชา

1. ในช่วง 12 เดือนที่ผ่านมาท่านได้ใช้กัญชาหรือไม่
   [ ] 0) ไม่ได้ใช้ [ ] 1) ใช้ ระบุจำนวนวันที่ใช้...................วัน

2. ท่านเริ่มนำกัญชามาใช้ครั้งแรกเมื่ออายุเท่าใด ....ปี

3. เหตุผลที่ท่านใช้กัญชาในครั้งแรก (ตอบได้ 1 ข้อ)
   [ ] 1. เพื่อผ่อนคลาย [ ] 2. ความอยากรู้อยากเห็น [ ] 3. เพื่อการเข้าสังคม การยอมรับจากเพื่อน
   [ ] 4. ได้รับอิทธิพลจากสื่อ [ ] 5. เหตุผลทางการแพทย์ [ ] 6. อื่น ๆ ระบุ..

4. ในรอบ 12 เดือนที่ผ่านมา ท่านใช้กัญชาด้วยวัตถุประสงค์ด้านใดบ้าง (ตอบได้**<u>มากกว่า</u>** 1 ข้อ)
   [ ] 1. เพื่อผ่อนคลาย [ ] 2. ความอยากรู้อยากเห็น [ ] 3. เพื่อการเข้าสังคม การยอมรับจากเพื่อน
   [ ] 4. ได้รับอิทธิพลจากสื่อ [ ] 5. เหตุผลทางการแพทย์ [ ] 6. อื่น ๆ ระบุ..

5. ในรอบ 12 เดือนที่ผ่านมา ท่านใช้กัญชาด้วยวัตถุประสงค์ใดมากที่สุด (ตอบได้ 1 ข้อ)
   [ ] 1. เพื่อผ่อนคลาย [ ] 2. ความอยากรู้อยากเห็น [ ] 3. เพื่อการเข้าสังคม การยอมรับจากเพื่อน
   [ ] 4. ได้รับอิทธิพลจากสื่อ [ ] 5. เหตุผลทางการแพทย์ [ ] 6. อื่น ๆ ระบุ..

ส่วนที่ 3 การใช้กัญชาทางการแพทย์

1. ท่านเริ่มนำใช้กัญชามาใช้เพื่อทางการแพทย์หรือเพื่อการรักษาโรค บรรเทาอาการเจ็บป่วยครั้งแรกเมื่ออายุ ....ปี
2. ท่านใช้กัญชาเพื่อวัตถุประสงค์ทางการแพทย์มาแล้วเป็นเวลานานเท่าใด ....ปี..............เดือน................วัน
3. เหตุใดท่านจึงตัดสินใจใช้กัญชาเพื่อรักษาโรคของท่าน ระบุ..

   ....

   ....
4. ปัจจุบันท่านใช้ยาแผนปัจจุบันอยู่ด้วยหรือไม่ [ ] 1) ใช้ [ ] 2) ไม่ใช้
5. ปัจจุบันท่านได้ลดหรือเลิกการใช้ยาแผนปัจจุบัน หลังจากที่ได้รับกัญชาทางการแพทย์หรือไม่

   [ ] 1) ใช่ โปรดอธิบายเหตุผล ....

   ....

   [ ] 2) ไม่ใช่ โปรดอธิบายเหตุผล...

   ....
6. ท่านเคยหยุดใช้กัญชาเพื่อการรักษาโรคหรือไม่ [ ] 1) ไม่เคยหยุด (ข้ามไปข้อ 6) [ ] 2) เคยหยุด

   เคยหยุด เพราะอะไร [ ] 1) ผิดกฎหมาย [ ] 2) แพงเกินไป [ ] 3) ไม่มั่นใจในคุณภาพของกัญชา

   [ ] 4) รู้สึกกดดันจากคนรอบข้าง [ ] 5) กัญชาไม่สามารถรักษาโรคที่กำลังเป็นอยู่ [ ] 6) ใช้แล้วรู้สึกผิด

   [ ] 7) แพทย์ไม่แนะนำให้ใช้ [ ] 8) แพทย์แนะนำให้ใช้ แต่ตัวเองคิดว่ายังไม่ต้องใช้

   [ ] 9) ไม่สามารถหากัญชามาใช้ได้ [ ] 9) อื่น ๆ.........................
7. ลักษณะการใช้กัญชาเพื่อการรักษาโรค ในช่วง 12 เดือนที่ผ่านมา (ให้ถามทีละโรค)

**ชื่อโรค/อาการป่วย**	1.<br>(ถ้าเป็นมะเร็ง ระบุอวัยวะด้วย)	2.<br>(ถ้าเป็นมะเร็ง ระบุอวัยวะด้วย)
**ผู้วินิจฉัย**	[ ] 1 ตัวเอง [ ] 2 แพทย์แผนปัจจุบัน<br>[ ] 3 แพทย์แผนไทย [ ] 4 คนอื่น	[ ] 1 ตัวเอง [ ] 2 แพทย์แผนปัจจุบัน<br>[ ] 3 แพทย์แผนไทย [ ] 4 คนอื่น ....
**ป่วยมานาน**	..............ปี...............เดือน...........................วัน	..............ปี...............เดือน...........................วัน
**เริ่มใช้ยากัญชา**	วันที่..............เดือน............................พ.ศ..................	วันที่..............เดือน............................พ.ศ..................
**รูปแบบของผลิตภัณฑ์กัญชาที่ใช้**	(ถ้าใช้น้ำมันสกัด ระบุชนิด THC/CBD และ % ความเข้มข้น)	(ถ้าใช้น้ำมันสกัด ระบุชนิด THC/CBD และ % ความเข้มข้น)
**วิธีใช้**		
**ความถี่ของการใช้**	[ ]1) หลายครั้งต่อวัน [ ]2) 6-7 วันต่อสัปดาห์<br>[ ]3) 3-5 วันต่อสัปดาห์ [ ]4) 1-2 วันต่อสัปดาห์<br>[ ]5) น้อยกว่าสัปดาห์ละครั้ง[ ]6) เดือนละ 1 ครั้ง<br>[ ]7) น้อยกว่าเดือนละ 1 ครั้ง)	1) หลายครั้งต่อวัน 2) 6-7 วันต่อสัปดาห์<br>3) 3-5 วันต่อสัปดาห์ 4) 1-2 วันต่อสัปดาห์<br>5) น้อยกว่าสัปดาห์ละครั้ง 6) เดือนละ 1 ครั้ง<br>7) น้อยกว่าเดือนละ 1 ครั้ง)
**ปริมาณที่ใช้ต่อครั้ง**	(จำนวน)...........................(หน่วย)..............................	(จำนวน)...........................(หน่วย)..............................
**ราคา**	หน่วยละ...........................บาท	หน่วยละ...........................บาท
**ซื้อ/ได้รับกัญชาจาก**	(ระบุผู้ขาย/ให้ยา/สถานที่โดยละเอียด)	(ระบุผู้จ่ายยา/สถานที่โดยละเอียด)
**ซื้อ/ได้รับครั้งแรกสุดเมื่อ**	วันที่...........เดือน.............................พ.ศ..................	วันที่...........เดือน.............................พ.ศ..................
**ซื้อ/ได้รับครั้งสุดท้ายเมื่อ**	วันที่...........เดือน.............................พ.ศ..................	วันที่...........เดือน.............................พ.ศ..................

**ครั้งล่าสุดที่ซื้อราคา**	(หน่วย)........................... ละ..............................บาท	(หน่วย)........................... ละ..............................บาท
**วิธีการซื้อ/ได้รับ**		
**หลังจากใช้อาการโรคเปลี่ยนไปอย่างไร**	[ ]1 ดีขึ้นมาก [ ]2 ดีขึ้น [ ]3 เหมือนเดิม [ ]4 แย่ลง [ ]5แย่ลงมาก	[ ]1 ดีขึ้นมาก [ ]2 ดีขึ้น [ ]3 เหมือนเดิม [ ]4 แย่ลง [ ]5แย่ลงมาก
**ปริมาณกัญชาที่ใช้เปลี่ยนแปลงไปหลังจากเริ่มใช้ครั้งแรกหรือไม่**	[ ]1 ใช้เพิ่มขึ้นมาก [ ]2 ใช้เพิ่มขึ้น [ ]3 ใช้เท่าเดิม [ ]4 ใช้น้อยลง [ ]5 ใช้น้อยลงมาก	[ ]1 ใช้เพิ่มขึ้นมาก [ ]2 ใช้เพิ่มขึ้น [ ]3 ใช้เท่าเดิม [ ]4 ใช้น้อยลง [ ]5 ใช้น้อยลงมาก

**ชื่อโรค/อาการป่วย**	3. (ถ้าเป็นมะเร็ง ระบุอวัยวะด้วย)	4. (ถ้าเป็นมะเร็ง ระบุอวัยวะด้วย)
**ผู้วินิจฉัย**	[ ] 1 ตัวเอง [ ] 2 แพทย์แผนปัจจุบัน [ ] 3 แพทย์แผนไทย [ ] 4 คนอื่น	[ ] 1 ตัวเอง [ ] 2 แพทย์แผนปัจจุบัน [ ] 3 แพทย์แผนไทย [ ] 4 คนอื่น ....
**ป่วยมานาน**	................ปี.................เดือน.............................วัน	................ปี.................เดือน.............................วัน
**เริ่มใช้ยากัญชา**	วันที่..............เดือน.............................พ.ศ...................	วันที่..............เดือน.............................พ.ศ...................
**รูปแบบของกัญชาที่ใช้**	(ถ้าใช้น้ำมันสกัด ระบุชนิด THC/CBD และ % ความเข้มข้น)	(ถ้าใช้น้ำมันสกัด ระบุชนิด THC/CBD และ % ความเข้มข้น)
**วิธีใช้**		
**ความถี่ของการใช้**	[ ]1) หลายครั้งต่อวัน [ ]2) 6-7 วันต่อสัปดาห์ [ ]3) 3-5 วันต่อสัปดาห์ [ ]4) 1-2 วันต่อสัปดาห์ [ ]5) น้อยกว่าสัปดาห์ละครั้ง[ ]6) เดือนละ 1 ครั้ง [ ]7) น้อยกว่าเดือนละ 1 ครั้ง)	1) หลายครั้งต่อวัน 2) 6-7 วันต่อสัปดาห์ 3) 3-5 วันต่อสัปดาห์ 4) 1-2 วันต่อสัปดาห์ 5) น้อยกว่าสัปดาห์ละครั้ง 6) เดือนละ 1 ครั้ง 7) น้อยกว่าเดือนละ 1 ครั้ง)
**ปริมาณ**	(จำนวน)............................(หน่วย)..............................	(จำนวน)............................(หน่วย)..............................
**ราคา**	หน่วยละ............................บาท	หน่วยละ............................บาท
**ซื้อ/ได้รับกัญชาจาก**	(ระบุผู้ขาย/ให้ยา/สถานที่โดยละเอียด)	(ระบุผู้จ่ายยา/สถานที่โดยละเอียด)
**ซื้อ/ได้รับครั้งแรกสุดเมื่อ**	วันที่............เดือน..............................พ.ศ...................	วันที่............เดือน..............................พ.ศ...................
**ซื้อ/ได้รับครั้งสุดท้ายเมื่อ**	วันที่............เดือน..............................พ.ศ...................	วันที่............เดือน..............................พ.ศ...................
**ครั้งล่าสุดที่ซื้อราคา**	(หน่วย)........................... ละ..............................บาท	(หน่วย)........................... ละ..............................บาท
**วิธีการซื้อ/ได้รับ**		
**หลังจากใช้อาการโรคเปลี่ยนไปอย่างไร**	[ ]1 ดีขึ้นมาก [ ]2 ดีขึ้น [ ]3 เหมือนเดิม [ ]4 แย่ลง [ ]5แย่ลงมาก	[ ]1 ดีขึ้นมาก [ ]2 ดีขึ้น [ ]3 เหมือนเดิม [ ]4 แย่ลง [ ]5แย่ลงมาก
**ปริมาณกัญชาที่ใช้เปลี่ยนแปลงไปหลังจากเริ่มใช้ครั้งแรกหรือไม่**	[ ]1 ใช้เพิ่มขึ้นมาก [ ]2 ใช้เพิ่มขึ้น [ ]3 ใช้เท่าเดิม [ ]4 ใช้น้อยลง [ ]5 ใช้น้อยลงมาก	[ ]1 ใช้เพิ่มขึ้นมาก [ ]2 ใช้เพิ่มขึ้น [ ]3 ใช้เท่าเดิม [ ]4 ใช้น้อยลง [ ]5 ใช้น้อยลงมาก

<u>รูปแบบของผลิตภัณฑ์กัญชาที่ใช้</u> 1. ดอก/ใบสด/แห้ง 2. น้ำมันสกัด 3. ซอฟเจล 4. ผง 5. ครีม/บาล์ม/ยาหม่องทาผิว 6. กาแฟ 7. คุกกี้/ขนม 8. สบู่ 9. ลิปสติก 10. สเปรย์ 11.อื่น ๆ ....

<u>วิธีใช้</u> 1. สูบ (ผสมบุหรี่ บ้องไอน้ำ/แห้ง) 2. กินเพียวหรือผสมน้ำ 3. หยดใต้ลิ้น 4. เหน็บ/สวนทวาร 5. บรรจุในแคปซูลและกิน 6. พ่น/ทา/อาบน้ำภายนอก 7. ต้มน้ำ/ชงดื่มเป็นชา 8.ใช้ Vaporization 9. อื่น ๆ ....

<u>วิธีการซื้อ/ได้รับยากัญชา</u> 1. สั่งซื้อจากเว็บไซต์ขายออนไลน์ 2. สั่งซื้อจากโซเชียลมีเดีย เช่น เฟสบุ๊ก line อินสตาแกรม เป็นต้น 3. แพทย์แผนปัจจุบันสั่งจ่ายให้ 4. แพทย์พื้นบ้าน/แผนไทยสั่งจ่ายให้ 5. เภสัชกร 6. บุคลากรสุขภาพอื่น ๆ.................... 7. ได้จากชมรม สมาคม เครือข่ายกลุ่มผู้ใช้กัญชา 8. หน่วยงานรัฐบาล 9. ร้านค้าในต่างประเทศ 10. ปลูก/ผลิตเอง 11. ผู้ขายยาในตลาดมืด 12. อื่นๆ ระบุ.......................

ส่วนที่ 4 การเข้าถึงกัญชา

1. ท่านได้รับข้อมูลข่าวสารกัญชาหรือสารสกัดของกัญชาได้จากที่ใด

[ ] 1. ชมรม สมาคม กลุ่มผู้ใช้กัญชา สมาพันธ์ ระบุ..

[ ] 2. จากโรงพยาบาล ระบุ..

[ ] 3. จากเว็บไซต์ขายออนไลน์ ระบุ..

[ ] 4. จากโซเชียลมีเดีย เช่น เฟซบุ๊ก line อินสตาแกรมเป็นต้น ระบุ..

....

[ ] 5.จากเพื่อน ระบุระดับความสัมพันธ์...

[ ] 6.จากผู้ขายที่ขาย ระบุ...

[ ] 7. จากหน่วยงานรัฐบาล ระบุ..

[ ] 8. อื่นๆ ระบุ...

**การรับรู้ต่อประโยชน์และโทษของการใช้กัญชา**	**ใช่**	**ไม่ใช่**
1. กัญชาสามารถรักษาอาการปวดเรื้อรังในผู้ใหญ่		
2. กัญชาเป็นยาต้านอาการอาเจียน ในการรักษาภาวะคลื่นไส้อาเจียนที่เกิดจากการได้รับเคมีบำบัด		
3. กัญชาช่วยทำให้อาการกล้ามเนื้อเกร็งลดลง		
4. กัญชาช่วยให้อาการนอนหลับดีขึ้นในผู้ที่มีปัญหาการนอนหลับ		
5. กัญชาเพิ่มความอยากอาหาร และลดการสูญเสียน้ำหนักในผู้ป่วย HIV/AIDS		
6. กัญชาช่วยทำให้อาการวิตกกังวลดีขึ้น		
7. กัญชาช่วยทำให้อาการของโรคเครียดหลังเผชิญภัยพิบัติดีขึ้น		
8. กัญชาช่วยรักษาโรคมะเร็ง		
9. กัญชาช่วยรักษาโรคเนื้องอกในสมอง		
10. กัญชาช่วยรักษาอาการผอมแห้งภาวะทุพโภชนาการจากโรคมะเร็ง		
11. กัญชาช่วยรักษาโรคลมชัก		
12. กัญชาช่วยรักษาโรคพาร์กินสัน		
13. กัญชาช่วยรักษาภาวะติดสารเสพติด		
14. กัญชาช่วยลดภาวะคลื่นไส้อาเจียนจากเคมีบำบัด		
15. กัญชาช่วยรักษาโรคลมชักรักษายากในเด็ก & โรคลมชักที่ดื้อต่อยารักษา		
16. กัญชาช่วยรักษาภาวะกล้ามเนื้อหดเกร็งในผู้ป่วยปลอกประสาทเสื่อมแข็ง		
17. กัญชาช่วยรักษาโรคอัลไซเมอร์		

การรับรู้ต่อประโยชน์และโทษของการใช้กัญชา	ใช่	ไม่ใช่
18. กัญชาช่วยรักษาโรคปลอกประสาทอักเสบ		
19. กัญชาช่วยรักษาโรควิตกกังวลไปทั่ว		
20. กัญชาช่วยลดอาการไอเรื้อรัง		
21. กัญชาทำให้เกิดภาวะหัวใจเต้นเร็วผิดปกติได้		
22. กัญชาทำให้เกิดภาวะตื่นตระหนก (แพนิก) ได้		
23. กัญชาทำให้เกิดภาวะสมองเสื่อม ความจำเสื่อม หมดแรงจูงใจในชีวิตได้		
24. กัญชาทำให้เกิดอาการโรคจิตคล้ายผู้ที่เป็นโรคจิตเภทได้		
25. กัญชาทำให้เกิดอาการปากแห้งอย่างรุนแรงได้		
26. กัญชาทำให้เกิดปฏิกิริยาการตอบสนองช้า ประสาทการเคลื่อนไหวผิดปกติ เพิ่มความเสี่ยงของการเกิดอุบัติเหตุ		
27. กัญชาทำให้เกิดภาวะประสาทหลอน		
28. กัญชาทำให้เกิดภาวะความดันโลหิตตกฉับพลัน		
29. กัญชาทำให้จำนวนเสปิร์มลดลง มีบุตรยาก		
30. กัญชาทำให้เดินเซ ควบคุมร่างกายได้ไม่ดีได้		
31. กัญชาทำให้เกิดอาการตาพร่ามัวได้		
32. กัญชาทำให้เกิดโรคตับอักเสบ		

ส่วนที่ 5 ความเห็นด้านการจัดการนโยบายกัญชาในอนาคตของประเทศไทย

คำถาม	เห็นด้วยอย่างยิ่ง	เห็นด้วย	เฉย ๆ	ไม่เห็นด้วย	ไม่เห็นด้วยอย่างยิ่ง
1 ประเทศไทยควรอนุญาตให้บุคคลที่บรรลุนิติภาวะแล้วสามารถใช้กัญชาเพื่อวัตถุประสงค์ทางการแพทย์ได้					
2 ผู้ใหญ่สามารถใช้กัญชาทางการแพทย์ได้แม้ว่าอยู่ต่อหน้าเด็กก็ตาม					
3 ถ้าผู้ปกครองใช้กัญชาทางการแพทย์จะเป็นการกระตุ้นให้เด็กอยากใช้กัญชามากขึ้น					
4 ประเทศไทยควรอนุญาตให้จำหน่ายผลิตภัณฑ์แปรรูปจากกัญชาใช้เพื่อการแพทย์ได้					

คำถาม	เห็นด้วยอย่างยิ่ง	เห็นด้วย	เฉย ๆ	ไม่เห็นด้วย	ไม่เห็นด้วยอย่างยิ่ง
5 ประเทศไทยควรอนุญาตให้จำหน่ายผลิตภัณฑ์แปรรูป จากกัญชาใช้เพื่อการผ่อนคลายได้					
6 ประเทศไทยควรอนุญาตประชาชนทั่วไป มีสิทธิในการ ปลูกกัญชา เพื่อวัตถุประสงค์ทางการแพทย์ได้					
7 ประเทศไทยควรอนุญาตให้ประชาชนทั่วไป มีสิทธิปลูกกัญชา เพื่อวัตถุประสงค์ในการผ่อนคลายได้					
8 อุตสาหกรรมกัญชาจะทำรายได้จำนวนมากให้กับประเทศ					
9 กัญชาควรเป็นสารเสพติดให้โทษทั้งในกรณีที่ใช้เพื่อการแพทย์ และ เหตุผลอื่น ดังที่เคยเป็นในอดีต (ก่อน พ.ศ. 2562)					
10 กัญชาควรมีกฎหมายควบคุมเช่นเดียวกับยาเสพติดรุนแรง เช่น ยาบ้า หรือ เฮโรอีน					
11กัญชาควรมีกฎหมายควบคุมเช่นเดียวกับเครื่องดื่ม แอลกอฮอล์					
12 กัญชาควรมีกฎหมายควบคุมเช่นเดียวกับยาสูบ บุหรี่					
