# Supplementary material for: Medical cannabis use in Thailand after its legalization: a respondent-driven sample survey
Source: PeerJ. 2022 Jan 11;10:e12809. doi: 10.7717/peerj.12809 (PMC8759353; doi:10.7717/peerj.12809)
Supplement: Supplemental Information 2 [file peerj-10-12809-s002.pdf]

Questionnaire

Current situation of medical cannabis use in Thailand

Number of medical cannabis users in your network...................................

Part 1 Demographic characteristics

1. Gender [ ] 1. male [ ] 2. female
2. Age …………...years 2.1 Date of birth (dd/mm/yy) …………………………………
3. Religion [ ] 1. Buddhist [ ] 2. Christian [ ] 3. Islam [ ] 4. Other (specify)………………………
4. Highest education attainment
   [ ] 1. not study [ ] 2. primary school [ ] 3. junior high school [ ] 4. Senior high school
   [ ] 5. vocational college [ ] 6. university or higher [ ] 7. Other, specify …………………
5. Main income earning occupation
   [ ] 1. Unemployed [ ] 2. Student [ ] 3. Labour worker [ ] 4.gardener/farmer/agricultural worker
   [ ] 5. entrepreneur [ ] 6. private business employee [ ] 7. government official
   [ ] 8. state enterprise employee [ ] 9. business owner
   [ ] 10. priest [ ] 11. Other, specify …………………………
6. Marital status
   [ ] 1.single [ ] 2.separated [ ] 3.widowed [ ] 4.divorced [ ] 5.married

7. Address, province ....

Part 2 Pattern of cannabis use

1. In the past 12 months, did you use cannabis?
   [ ] 0 No [ ] 1 Yes, days of use…………………….
2. At what age did you start using cannabis? …………. years
3. Reasons for first use (can choose >1 item):
   [ ]1 for relaxation [ ]2 curiosity [ ]3 socialization [ ]4 media influence
   [ ]5 medical purpose [ ] 6. Other, specify...
4. In the past 12 months, what were the purposes of your use of cannabis (can choose >1)
   [ ]1 for relaxation [ ]2 curiosity [ ]3 socialization [ ]4 media influence
   [ ]5 medical purpose [ ] 6. Other, specify...
5. In the past 12 months, what was the most common reason for your use of cannabis?:
   [ ]1 for relaxation [ ]2 curiosity [ ]3 socialization [ ]4 media influence
   [ ]5 medical purpose [ ] 6. Other, specify...

Part 3 Medical cannabis use

1. At what age did you start using cannabis for medical purpose? …………. years
2. How long have you been using cannabis for medical purpose? ....years...............months.................days
3. Why did you decide to use cannabis for medical reason?...
   ....
   ....
4. Do you currently use modern medicine? [ ] 1) yes [ ] 2) no
5. Have you reduce or stop using modern medicine after using medical cannabis?
   [ ] 1) yes, why? ....
   ....

[ ] 2) no, why?..

....

6. Have you ever stopped using medical cannabis? [ ] 1) no (skip to 6) [ ] 2) yes

Why stopped [ ] 1) illegal [ ] 2) too expensive [ ] 3) not trust its quality

[ ] 4) pressure from other people [ ] 5) cannabis cannot treat the present illness

[ ] 6) feel guilty [ ] 7) doctor advised not to use [ ] 8) doctor advised to use but I think it's not necessary [ ] 9) cannot find cannabis to use [ ] 9) Other, specify.........................

7. Pattern of medical cannabis use in the past 12 months (asking one by one disease)

**Disease/symptom**	1.<br>(if CA, specify organ)	2.<br>(if CA, specify organ)
**Who diagnosed?**	[ ] 1 self [ ] 2 doctor<br>[ ] 3 TM doctor<br>[ ] 4 other ………………..	[ ] 1 self [ ] 2 doctor<br>[ ] 3 TM doctor<br>[ ] 4 other ………………..
**For how long**	................y.................m.............................d	................y.................m.............................d
**Started cannabis**	date...............mo...................B.C.............	date...............mo..............B.C.....................
**Form of cannabis**	(for oil specify THC/CBD and % conc.)	(for oil specify THC/CBD and % conc.)
**Method of use**		
**Frequency**	[ ]1) many times/d [ ]2) 6-7 d/w<br>[ ]3) 3-5 d/w [ ]4) 1-2 d/w<br>[ ]5) ,1 d/w [ ]6) 1/month [ ]7) <1/month	[ ]1) many times/d [ ]2) 6-7 d/w<br>[ ]3) 3-5 d/w [ ]4) 1-2 d/w<br>[ ]5) ,1 d/w [ ]6) 1/month [ ]7) <1/month
**Quantity/time**	(Quantity).............................(Unit)................	(Quantity).............................(Unit)................
**Price**	.............................Baht/unit	.............................Baht/unit
**Bought/got from**	(give detail)	(give detail)
**First use**	date...............mo............B.C.....................	date...............mo...............B.C.....................
**Latest buy**	date...............mo..............B.C.....................	date...............mo...............B.C.....................
**Latest price**	(Unit)......................p..............................Baht	(Unit)......................p..............................Baht
**Method of buying**		
**Change of disease after use**	[ ]1 very much better [ ]2 better<br>[ ]3 same<br>[ ]4 worse [ ]5 very much worse	[ ]1 very much better [ ]2 better<br>[ ]3 same<br>[ ]4 worse [ ]5 very much worse
**Change of quantity after first use**	[ ]1 very much increase<br>[ ]2 increase [ ]3 same<br>[ ]4 decrease [ ]5 very much decrease	[ ]1 very much increase<br>[ ]2 increase [ ]3 same<br>[ ]4 decrease [ ]5 very much decrease

Disease/symptom	3. (if CA, specify organ)	4. (if CA, specify organ)
Who diagnosed?	[ ] 1 self [ ] 2 doctor<br>[ ] 3 TM doctor<br>[ ] 4 other ………………..	[ ] 1 self [ ] 2 doctor<br>[ ] 3 TM doctor<br>[ ] 4 other ………………..
For how long	................y.................m............................d	................y.................m............................d
Started cannabis	date...............mo................B.C....................	date...............mo................... B.C...........
Form of cannabis	(for oil specify THC/CBD and % conc.)	(for oil specify THC/CBD and % conc.)
Method of use		
Frequency	[ ]1) many times/d [ ]2) 6-7 d/w<br>[ ]3) 3-5 d/w [ ]4) 1-2 d/w<br>[ ]5) ,1 d/w [ ]6) 1/month [ ]7) <1/month	[ ]1) many times/d [ ]2) 6-7 d/w<br>[ ]3) 3-5 d/w [ ]4) 1-2 d/w<br>[ ]5) ,1 d/w [ ]6) 1/month [ ]7) <1/month
Quantity/time	(Quantity).....................(Unit).....................	(Quantity)..................(Unit)........
Price	.............................Baht/unit	.............................Baht/unit
Bought/got from	(give detail)	(give detail)
First use	date...............mo................B.C....................	date...............mo................B.C....................
Latest buy	date...............mo................B.C....................	date...............mo................B.C....................
Latest price	(Unit)............... p..............................Baht	(Unit)................ p..............................Baht
Method of buying		
Change of disease after use	[ ]1 very much better [ ]2 better<br>[ ]3 same<br>[ ]4 worse [ ]5 very much worse	[ ]1 very much better [ ]2 better<br>[ ]3 same<br>[ ]4 worse [ ]5 very much worse
Change of quantity after first use	[ ]1 very much increase<br>[ ]2 increase [ ]3 same<br>[ ]4 decrease [ ]5 very much decrease	[ ]1 very much increase<br>[ ]2 increase [ ]3 same<br>[ ]4 decrease[ ]5 very much decrease

Form of cannabis product 1. Flower/leaf/root 2. Oil extract 3. Soft gel 4. powder 5. Cream/ba 6. coffee 7. Cookie/cake 8. soap 9. lipstick 10. spray 11. Other, specify ....

Method of use 1. smoke (mix in cigarette, joint) 2. Drink pure or mixed with water 3. sublingual 4. Rectal suppository 5. Put in capsule and eat 6. Spray/topical put on 7. Drink as tea 8. Vaporization 9. Other, specify ....

Method of buying/obtaining 1. Order from website 2. Order from social media 3. Doctor prescribed 4. TM doctor/folk healer 5. pharmacist 6. Other healthcare worker..................... 7. Network, association, user group

8. Government organization 9. Overseas shop 10. Home grown/production 11. Underground dealer
12. Other, specify.......................

Part 4 Perception of benefits and harms

1. From where did you receive information about medical cannabis?

[ ] 1. Network, association, group, specify..

[ ] 2. Hospital, specify...

[ ] 3. Sale website, specify...

[ ] 4. Social media. ....

[ ] 5.friend...

[ ] 6.seller...

[ ] 7. government organization...

[ ] 8. Other, specify...

**Perception of benefit and harm: Cannabis can…**	ใช่	ไม่ใช่
1. Treat chronic pain in adults		
2. An antiemesis for patients who receive chemotherapy		
3. Improve spasticity		
4. Improve insomnia		
5. Increase appetite in HIV/AIDS patients		
6. Improve anxiety symptoms		
7. Improve PTSD symptoms		
8. Treat cancers		
9. Treat brain tumour		
10. Treat anorexia, malnutrition from cancer		
11. Treat epilepsy		
12. Treat Parkinson's disease		
13. Treat substance dependence		
14. Reduce nausea and vomiting		
15. Treat intractable epilepsy in children and treatment resistant epilepsy		
16. Treat spasticity form multiple sclerosis		
17. Treat Alzheimer's disease		
18. Treat multiple sclerosis		

Perception of benefit and harm: Cannabis can…	ใช่	ไม่ใช่
19.Treat generalized anxiety disorder		
20. Decrease severity of chronic cough		
21. Cause Palpitations		
22. Cause Panic symptoms		
23. Cause Dementia, memory impairment, amotivation		
24. Cause Schizophrenia-like psychotic symptoms		
25. Cause Severe dry mouth		
26. Cause Slow reaction time, abnormal sensory-motor function		
27. Cause Hallucinations		
28. Cause Acute hypotension		
29. Cause Decreased sperm count, infertility		
30. Cause Ataxia, uncontrollable body coordination		
31. Cause Blurred vision		
32. Cause Hepatitis		

Part 5: Opinion towards cannabis policy in Thailand

Statement	Strongly agree	agree	neutral	disagree	Strongly disagree
1 Thailand should allow people over 20 years old use cannabis for medical purpose.					
2 Adults can use cannabis even being in front of children					
3 If parents use cannabis it will stimulate children to use it.					
4 Thailand should allow selling cannabis products for medical purpose.					
5 Thailand should allow selling cannabis products for recreational purpose.					
6 Thailand should allow people grow cannabis for medical purpose.					

**Statement**	Strongly agree	agree	neutral	disagree	Strongly disagree
7 Thailand should allow people grow cannabis for recreational purpose.					
8 Cannabis industry will produce large income to the country					
9 Cannabis should remain drug of abuse either for medical or recreational use as that in the past (before legalization in 2019)					
10 Cannabis should be under a strict law at the same level as heroin and methamphetamine					
11 Cannabis should be under a control law at the same level as alcohol					
12 Cannabis should be under a control law at the same level as tobacco					
